# Scanning barcodes: A way to explore viral populations

**DOI:** 10.1371/journal.ppat.1011291

**Published:** 2023-04-20

**Authors:** Emily A. Fitzmeyer, Emily N. Gallichotte, Gregory D. Ebel

**Affiliations:** Center for Vector-borne Infectious Diseases, Department of Microbiology, Immunology and Pathology, Colorado State University, Fort Collins, Colorado, United States of America; Mount Sinai School of Medicine, UNITED STATES

Due to error-prone replication, RNA viruses such as Zika virus (ZIKV), West Nile virus (WNV), influenza A virus (IAV), and simian and human immunodeficiency viruses (SIV and HIV, respectively) exist in nature as genetically and phenotypically complex mutant swarms [1–3]. The ability of RNA viruses to be maintained in nature as a mutant swarm is thought to promote adaptive plasticity and facilitate the evolution and emergence of these viruses [3]. Investigating swarm dynamics during infection, transmission, and treatment is therefore of great significance. Studying intrahost virus population dynamics typically requires identifying intrahost single nucleotide variants (iSNVs) using various approaches to whole-genome sequencing [4]. While these approaches are well suited to exploring virus diversification and measuring how natural selection shapes the virus genome, they are not well suited to quantitatively assess reductions in virus diversity. Barcoded viruses are a rapidly expanding technology that allows researchers to quantitatively characterize aspects of virus population dynamics with greater sensitivity and resolution than can be achieved with computational haplotype reconstruction [5–7].

## What is a barcoded virus?

**Fig 1:**
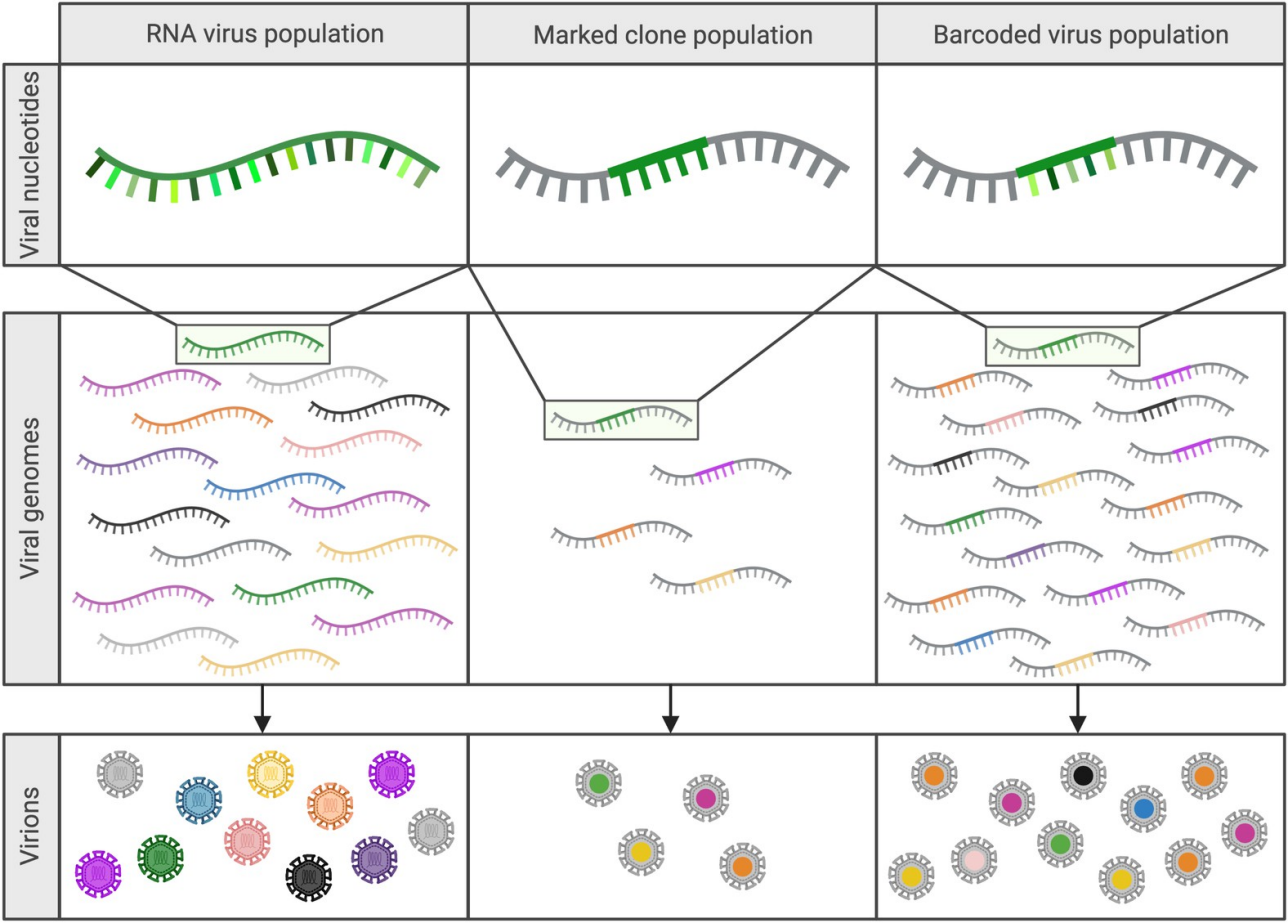
Barcoded viruses are highly effective mimics of RNA virus mutant swarms. Marked clones and barcoded viruses allow investigators to study changes to population diversity in a variety of systems without the variable fitness observed between viruses in natural RNA virus swarms. (Created with Biorender.com).

A barcoded virus is engineered to contain a sequence motif (i.e., a barcode) that can be used to distinguish one otherwise identical virus genome from another (**Fig 1**). While technical approaches to generating barcoded virus populations vary by virus and study, barcoded viruses generally contain a series of coding-neutral alterations to the genome [7–9]. Barcodes commonly range from 9 to >40 nucleotides in length and are either placed in a noncoding region of the genome (e.g., 3′ or 5′ untranslated regions) or into coding sequences that contain a series of adjacent codons with fully synonymous third positions (i.e., Leu, Val, Ser, Pro, Thr, and Ala). The barcodes are introduced to the viral genome via site-directed mutagenesis (SDM) or custom synthetic gene fragments [2,7,10]. Early iterations of barcoded viruses were marked clones, the marked (i.e., barcoded) regions of which were introduced individually by methods such as SDM [2,11,12]. Due to the constraints associated with developing each unique virus individually, marked clone populations often contain a small fraction—less than 20 unique clones—of the diversity that can now be achieved in barcoded virus populations [2,11] (**Fig 1**). Current applications of barcoded virus technology use degenerate nucleotides that allow all possible mutations to occur at the site of insertion and, upon amplification and rescue of the recombinant virus, can theoretically generate several million unique barcodes without the burden of generating clones individually [7,8]. Due to the nature of barcode generation, this technology is currently limited to viruses for which efficient reverse genetics, cloning, and rescue systems already exist.

## What is the appeal of using a barcoded virus population to study population dynamics over traditional haplotype reconstruction?

Uniquely barcoded viruses within a population function as analogues for naturally occurring variant genomes within a mutant swarm, making them powerful tools for mimicking RNA virus populations [3,9]. Barcoded virus populations are high-diversity representations of virus populations and thus allow for a more precise characterization of population dynamics than can be achieved by characterizing naturally occurring diversity [5,7,10]. Since barcodes are introduced to the virus genome without altering the coding sequence, they theoretically have a minimal impact on fitness. This distinguishes them from true quasispecies virus populations, which contain naturally occurring mutations that may confer significant phenotypic variability [3]. Defining natural variants requires whole-genome sequencing and is typically accomplished using short-read sequencing, and assigning reads to individual genomes (binning) to identify variants. This method of variant identification, when employed to quantify population diversity, is extremely sensitive to errors introduced during sample preparation and sequencing [13].

While the fitness-neutral nature of the barcode population model precludes it from capturing the full range of evolutionary processes (such as positive selection), stochastic forces, such as bottlenecks, have a significant impact on virus evolution within individual hosts [14]. Barcoded viruses are extremely well suited to quantifying these stochastic forces shaping virus populations, as the neutrality of the barcode allows investigators to examine population dynamics without the variable of fitness [2,9–11]. Barcoded viruses also provide solutions for examining infection dynamics in systems where intrahost virus diversity is typically constrained or too low to uniquely identify viral lineages, or where independent tracking of multiple infection events, which would be difficult to track by conventional methods, is required [7,15]. Additionally, the function of barcodes as unique identifiers allows for determination of the clonal origin of viruses throughout infection and allows for the identification and quantification of variant analogues within a population with extremely high resolution and sensitivity [5–7,16]. Finally, using barcode-specific probes, the population dynamics of barcoded viruses can be tracked during transmission events and analyzed by quantitative reverse transcription PCR (qRT-PCR) without the need for deep-sequencing [11].

## How has barcoded virus technology impacted the field?

The predominant use of barcoded viruses is as mimics of highly diverse virus populations in studies of population dynamics during infection, transmission, and treatment. Early work successfully demonstrated the value of synthetic swarm viruses as analogues for virus populations by using marked clone populations to characterize the impact of stochastic forces, such as bottlenecks (i.e., random and rapid reduction of diversity in a virus population), on poliovirus populations during neuroinvasion in mice, and on WNV and Venezuelan equine encephalitis virus (VEEV) populations within relevant mosquito vectors [2,11,12]. This work shed light on the adaptive potential of these pathogens, highlighted the important role infection plays in shaping virus populations, and established synthetic swarms as powerful molecular tools [2,11,12]. Since this foundational work with poliovirus, WNV, and VEEV, barcoded viruses have been utilized to answer questions about population dynamics across numerous virus families.

**Fig 2:**
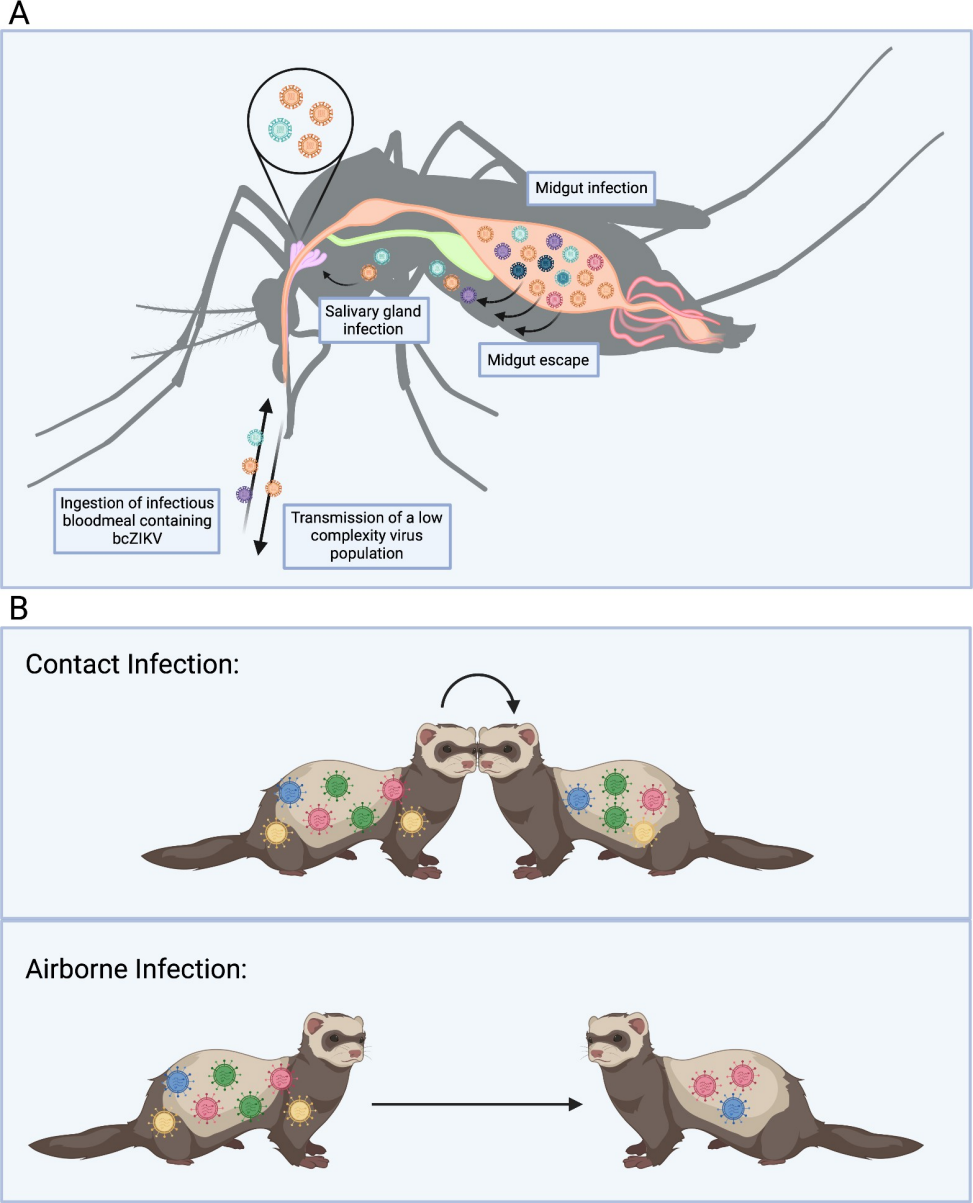
Barcoded viruses mimic virus population dynamics during transmission and infection events. **(A)** Intrahost bottlenecks in *Ae*. *aegypti* rapidly reduce the complexity of a bcZIKV population during mosquito infection. (**B)** A more complex bcIAV population is transmitted between ferrets via contact infection than airborne infection. (Created with Biorender.com)

*bcZIKV*. Barcoded Zika virus (bcZIKV) was used to characterize ZIKV infection dynamics in pregnant and nonpregnant macaques [15]. Low complexity barcode populations persisted in pregnant animals after typical resolution of infection in nonpregnant animals, indicating that an anatomical reservoir had been established in the pregnant macaques [15]. This work provided proof-of-concept for the use of bcZIKV *in vivo* to examine virus populations throughout infection and highlighted the potential of barcoded viruses to probe the impacts of anatomical reservoirs, and bottlenecks on virus populations. bcZIKV also identified a cumulative reduction in bcZIKV population diversity associated with intrahost bottlenecks during infection in *Aedes aegypti* mosquitoes [8] (**Fig 2A**). bcZIKV has also been instrumental in determining the impact of transmission modes between vertebrates and mosquitoes on ZIKV evolution and was used to demonstrate how direct vertebrate transmission chains could promote enhanced ZIKV virulence [17]. Further, bcZIKV was used to identify diversity in individual plaque forming units, demonstrating that ZIKV could potentially be transmitted as multigenome aggregates [18].

*bcIAV*. Barcoded influenza A virus (bcIAV) has been employed to study infection routes and their associated bottlenecks, evolution of the NS1 gene, virus replication in different regions of the respiratory tract, and the impact of compartmentalized replication on virus population dynamics [1,9,10,19]. Studies of bcIAV in ferrets allowed for a high-resolution characterization of physiological bottlenecks and demonstrated that aerosol transmission represents a severe and highly restrictive bottleneck [9] (**Fig 2B**). Additionally, bcIAV infection of ferret lungs revealed that a series of bottlenecks within the lungs results in genetically distinct virus population “islands” that are heavily impacted by founder effects [9,10]. Further work with bcIAV in the respiratory tract established that transmissible droplets are generated in upper respiratory tissues, providing an anatomical target for viral load reduction to prevent IAV transmission [19]. bcIAV has also been instrumental in demonstrating the adaptive plasticity of the IAV NS1 gene, and in quantifying reassortment of IAV upon multiple infection [1,20].

*bcCVB*. Coxsackievirus B3 (CVB3) is an enterovirus that can penetrate the gastrointestinal tract and cause systemic infection [21]. A highly rich barcoded CVB3 (bcCVB3) population was used to study CVB3 population dynamics in mice and allowed researchers to quantify the impact of the gastrointestinal (GI) barrier on a CVB3 population, demonstrating a significant reduction in barcode diversity upon infection of and replication in extraintestinal tissues [21].

*bcSIV* and *bcHIV*. Barcoded SIV and HIV (bcSIV and bcHIV, respectively) have been used in numerous studies to explore retrovirus population dynamics during transmission, infection, immune escape, treatment, and reactivation and rebound following latency [5–7,16,22–25]. Using bcSIV, investigators have shown that intrarectal (IR) inoculation of macaques results in 70- to 560-fold less complex SIV populations when compared to intravenous inoculation, demonstrating that infection barriers associated with IR challenge impose a population bottleneck on SIV populations [25]. Further studies with bcSIV quantified the impact of the host immune response on an SIV population and the level of replication preceding the generation of escape mutations in unique lineages within an SIV population [6]. Studies of reactivation and rebound following latency or the interruption of combination antiretroviral therapy (cART) with bcSIV established an estimated rate of reactivation in viral reservoirs, an approximate viral load per reactivated latent cell, and determined that the viral lineages that were dominant in the population pretreatment tend to reactivate first during treatment interruption [7,22,23]. Finally, studies of bcHIV populations in mice revealed the efficacy of latency-reversing agents (LRAs) in reducing the diversity of rebound populations and delaying rebound upon interruption of cART [16].

*bcAAV*. Adeno-associated viral (AAV) vectors are a clinically relevant mode of therapeutic gene transfer that have had success in reprogramming certain cell types in animal models [26]. Given the high demand for this therapeutic, there is a growing need to develop and screen AAVs for increased or tissue-specific transduction efficiency [26–29]. Barcoded AAVs (bcAAV) are frequently employed as high-throughput screening tools for recombinant AAV vector pools that allow investigators to quantify multiple AAV genome and transcript abundances in parallel in tissues of interest [27,29].

## Why should I care about barcodes?

The barcoded virus approach to studying virus evolution is reliable, allows for investigation of population dynamics with unprecedented depth and ease, and has been successfully adapted to study these phenomena in a wide range of virus families and hosts. Barcoded viruses have proven to be valuable tools in assessing viral replication dynamics, progeny production, and polyinfection (i.e., infection of a single cell with multiple unique genomes) at the single-cell level [30,31]. Finally, barcoded virus technology has the potential to be highly valuable for computational modeling of infection by providing quantitative estimates of host- and environment-dependent patterns of genetic restriction.

## References

[1] Muñoz-Moreno R, Martínez-Romero C, Blanco-Melo D, Forst Cv, Nachbagauer R, Benitez AA, et al. Viral Fitness Landscapes in Diverse Host Species Reveal Multiple Evolutionary Lines for the NS1 Gene of Influenza A Viruses. Cell Rep. 2019 Dec 17;29(12):3997–4009.e5. doi: 10.1016/j.celrep.2019.11.070 31851929 PMC7010214

[2] Ciota AT, Ehrbar DJ, van Slyke GA, Payne AF, Willsey GG, Viscio RE, et al. Quantification of intrahost bottlenecks of West Nile virus in Culex pipiens mosquitoes using an artificial mutant swarm. Infect Genet Evol. 201 Apr 2;12(3):557–564. 22326536 10.1016/j.meegid.2012.01.022 PMC3314143

[3] Domingo E, Holland JJ. RNA virus mutations and fitness for survival [internet]. 1997. Available from: www.annualreviews.org. doi: 10.1146/annurev.micro.51.1.151 9343347

[4] Grubaugh ND, Weger-Lucarelli J, Murrieta RA, Fauver JR, Garcia-Luna SM, Prasad AN, et al. Genetic Drift during Systemic Arbovirus Infection of Mosquito Vectors Leads to Decreased Relative Fitness during Host Switching. Cell Host Microbe. 2016 Apr 13;19(4):481–492. doi: 10.1016/j.chom.2016.03.002 27049584 PMC4833525

[5] Khanal S, Fennessey CM, O’Brien SP, Thorpe A, Reid C, Immonen TT, et al. In Vivo Validation of the Viral Barcoding of Simian Immunodeficiency Virus SIVmac239 and the Development of New Barcoded SIV and Subtype B and C Simian-Human Immunodeficiency Viruses. J Virol. 2019 Dec 12;94(1). doi: 10.1128/JVI.01420-19 31597757 PMC6912102

[6] Immonen TT, Camus C, Reid C, Fennessey CM, del Prete GQ, Davenport MP, et al. Genetically barcoded SIV reveals the emergence of escape mutations in multiple viral lineages during immune escape. Available from: www.pnas.org/cgi/doi/10.1073/pnas.1914967117. 10.1073/pnas.1914967117 PMC6955354 31843933

[7] Fennessey CM, Pinkevych M, Immonen TT, Reynaldi A, Venturi V, Nadella P, et al. Genetically-barcoded SIV facilitates enumeration of rebound variants and estimation of reactivation rates in nonhuman primates following interruption of suppressive antiretroviral therapy. PLoS Pathog. 2017 May 1;13(5). doi: 10.1371/journal.ppat.1006359 28472156 PMC5433785

[8] Weger-Lucarelli J, Garcia SM, Rückert C, Byas A, O’Connor SL, Aliota MT, et al. Using barcoded Zika virus to assess virus population structure in vitro and in Aedes aegypti mosquitoes. Virology. 2018 Aug 1(521):138–148. 10.1016/j.virol.2018.06.004 PMC6309320 29935423

[9] Varble A, Albrecht RA, Backes S, Crumiller M, Bouvier NM, Sachs D, et al. Influenza a virus transmission bottlenecks are defined by infection route and recipient host. Cell Host Microbe. 2014 Nov 12;16(5):691–700. doi: 10.1016/j.chom.2014.09.020 25456074 PMC4272616

[10] Amato KA, Haddock LA, Braun KM, Meliopoulos V, Livingston B, Honce R, et al. Influenza A virus undergoes compartmentalized replication in vivo dominated by stochastic bottlenecks. Nat Commun. 2022 Dec 1;13(1). doi: 10.1038/s41467-022-31147-0 35701424 PMC9197827

[11] Forrester NL, Guerbois M, Seymour RL, Spratt H, Weaver SC. Vector-Borne Transmission Imposes a Severe Bottleneck on an RNA Virus Population. PLoS Pathog 2012 Sep;8(9). 10.1371/journal.ppat.1002897 PMC3441635 23028310

[12] Pfeiffer JK, Kirkegaard K. Bottleneck-mediated quasispecies restriction during spread of an RNA virus from inoculation site to brain. Proc Natl Acad Sci U S A. 2006 Apr 4;103(14):5520–5525. doi: 10.1073/pnas.0600834103 16567621 PMC1414638

[13] McCrone JT, Lauring AS. Measurements of Intrahost Viral Diversity Are Extremely Sensitive to Systematic Errors in Variant Calling. J Virol 2016 Aug;90(15):6884–6895. doi: 10.1128/JVI.00667-16 27194763 PMC4944299

[14] Mccrone JT, Woods RJ, Martin ET, Malosh RE, Monto AS, Lauring AS. Stochastic processes constrain the within and between host evolution of influenza virus. doi: 10.7554/eLife.35962 29683424 PMC5933925

[15] Aliota MT, Dudley DM, Newman CM, Weger-Lucarelli J, Stewart LM, Koenig MR, et al. Molecularly barcoded Zika virus libraries to probe in vivo evolutionary dynamics. PLoS Pathog. 2018 Mar 1;14(3). doi: 10.1371/journal.ppat.1006964 29590202 PMC5891079

[16] Marsden MD, Zhang T-h, Du Y, Dimapasoc M, MSA S, Wu X, et al. Tracking HIV Rebound following Latency Reversal Using Barcoded HIV. Cell Rep Med. 2020 Dec 22;1(9). doi: 10.1016/j.xcrm.2020.100162 33377133 PMC7762775

[17] Riemersma KK, Jaeger AS, Crooks CM, Braun KM, Weger-Lucarelli J, Ebel GD, et al. Rapid Evolution of Enhanced Zika Virus Virulence during Direct Vertebrate Transmission Chains. J Virol. 2021. doi: 10.1128/JVI.02218-20 33536175 PMC8103699

[18] Sexton NR, Bellis ED, Murrieta RA, Spangler MC, Cline PJ, Weger-Lucarelli J, et al. Genome Number and Size Polymorphism in Zika Virus Infectious Units [Internet]. 2021. doi: 10.1128/JVI.00787-20 33328311 PMC8094933

[19] Xie C, Su W, Sia SF, Choy KT, Morrell S, Zhou J, et al. A(H1N1)pdm09 Influenza Viruses Replicating in Ferret Upper or Lower Respiratory Tract Differed in Onward Transmission Potential by Air. J Infect Dis. 2022 Jan 5;225(1):65–74. doi: 10.1093/infdis/jiab286 34036370 PMC8730494

[20] Phipps KL, Ganti K, Jacobs NT, Lee CY, Carnaccini S, White MC, et al. Collective interactions augment influenza A virus replication in a host-dependent manner. Nat Microbiol 2020 Sep 1;5 (9):1158–1169. doi: 10.1038/s41564-020-0749-2 32632248 PMC7484227

[21] Mccune BT, Lanahan MR, Tenoever BR, Pfeiffer JK. Rapid Dissemination and Monopolization of Viral Populations in Mice Revealed Using a Panel of Barcoded Viruses. 2020. doi: 10.1128/JVI PMC6955244 31666382

[22] Immonen TT, Fennessey CM, Lipkey L, Thorpe A, del Prete GQ, Lifson JD, et al. Transient viral replication during analytical treatment interruptions in SIV infected macaques can alter the rebound-competent viral reservoir. PLoS Pathog. 2021 Jun 1;17(6). doi: 10.1371/journal.ppat.1009686 34143853 PMC8244872

[23] Pinkevych M, Fennessey CM, Cromer D, Tolstrup M, Søgaard OS, Rasmussen TA, et al. Estimating Initial Viral Levels during Simian Immunodeficiency Virus/Human Immunodeficiency Virus Reactivation from Latency. J Virol 2018 Jan 15;92(2). doi: 10.1128/JVI.01667-17 29118123 PMC5752936

[24] Chen HC, Zorita E, Filion GJ. Using Barcoded HIV Ensembles (B-HIVE) for Single Provirus Transcriptomics. Curr Protoc Mol Biol. 2018 Apr 1;122(1). doi: 10.1002/cpmb.56 29851299

[25] Moriarty R, Golfinos AE, Gellerup DD, Schweigert H, Mathiaparanam J, Balgeman AJ, et al. The mucosal barrier and anti-viral immune responses can eliminate portions of the viral population during transmission and early viral growth. PLoS ONE. 2021 Dec 1;16(12 December). doi: 10.1371/journal.pone.0260010 34855793 PMC8639003

[26] Pekrun K, de Alencastro G, Luo QJ, Liu J, Kim Y, Nygaard S, et al. Using a barcoded AAV capsid library to select for clinically relevant gene therapy vectors JCI Insight. 2019 Nov 14;4(22). 10.1172/jci.insight.131610 PMC6948855 31723052

[27] Weinmann J, Weis S, Sippel J, Tulalamba W, Remes A, el Andari J, et al. Identification of a myotropic AAV by massively parallel in vivo evaluation of barcoded capsid variants. Nat Commun. 2020 Dec 1;11(1). doi: 10.1038/s41467-020-19230-w 33116134 PMC7595228

[28] Brown D, Altermatt M, Dobreva T, Chen S, Wang A, Thomson M, et al. Deep Parallel Characterization of AAV Tropism and AAV-Mediated Transcriptional Changes via Single-Cell RNA Sequencing. Front Immunol. 2021 Oct 21:12. doi: 10.3389/fimmu.2021.730825 34759919 PMC8574206

[29] Xu M, Li J, Xie J, He R, Su Q, Gao G, et al. High-Throughput Quantification of In Vivo Adeno-Associated Virus Transduction with Barcoded Non-Coding RNAs. Hum Gene Ther. 2019 Aug 1;30(8):946–956. doi: 10.1089/hum.2018.253 31072208 PMC6703241

[30] Frank DT, Byas AD, Murrieta R, Weger-Lucarelli J, Rückert C, Gallichotte E, et al. Intracellular diversity of WNV within circulating avian peripheral blood mononuclear cells reveals host-dependent patterns of polyinfection. doi: 10.1101/2023.01.27.525959 37375457 PMC10300861

[31] Bacsik DJ, Dadonaite B, Butler A, Greaney AJ, Heaton NS, Bloom JD, et al. Influenza virus transcription and progeny production are poorly correlated in single cells. doi: 10.1101/2022.08.30.505828 PMC10484525 37675839

